# Podoconiosis instruction at nursing schools in Kenya, Rwanda, and Uganda

**DOI:** 10.1186/s41182-022-00405-8

**Published:** 2022-02-11

**Authors:** Lilian Nantume Wampande, Lamek Mageto Nyabuga, Kelly Fowler, Grace Chinelo Okengwu, Ursin Bayisenge, Janna M. Schurer

**Affiliations:** 1 Center for One Health, University of Global Health Equity, Butaro, Rwanda; 2 Department of Global Health and Infectious Disease, Cummings School of Veterinary Medicine, North Grafton, USA

**Keywords:** Africa, Lymphedema, Nursing education, Podoconiosis, Neglected tropical disease

## Abstract

**Background:**

Podoconiosis is a preventable, progressive, and non-infectious form of elephantiasis that can contribute to significant disability and economic burden when not treated early. Nurses play a critical role in early detection and response in rural Africa, but it is unclear if they receive adequate training on podoconiosis. We aimed to characterize podoconiosis instruction at all government accredited, post-secondary nursing institutions in three African countries.

**Methods:**

Data for this cross-sectional study was collected through a quantitative survey with several open-answer questions. Through a rigorous online search, we identified all post-secondary institutions in Kenya, Rwanda and Uganda accredited to teach human nursing. A total of 289 accredited programs, including 85 certificate, 56 degree and 148 diploma programs were invited to participate. Respondents completed surveys online or by telephone. Measures focused on podoconiosis knowledge, perceptions of quality/quantity of podoconiosis instruction, and barriers to sufficient podoconiosis education.

**Results:**

We obtained information about 212 curricula across 149 nursing institutions in the three countries (participation rate: 73.4%). Podoconiosis coverage was limited across programs (certificate—24.1%; diploma—55.6%; degree—30.3%). Most respondents felt that the quality and quantity of instruction were insufficient (60.6%, 62.9%), respectively. Exclusion from government curricula, low priority and faculty lack of knowledge were commonly reported barriers to podoconiosis inclusion.

**Conclusions:**

This study demonstrated clear gaps in podoconiosis training for nurses across the three countries and highlights a serious challenge in eliminating podoconiosis as a public health problem. Interventions to improve nurses’ knowledge could include the development and free distribution of podoconiosis teaching materials, designed for integration into pre-existing courses.

## Background

Podoconiosis is one of 20 Neglected Tropical Diseases (NTDs) recognized by the World Health Organization (WHO) [1]. It is a chronic, disfiguring, and non-infectious type of elephantiasis that predominantly affects individuals marginalized by poverty [2]. The disease is caused by prolonged contact with irritant clay soils and is one of the leading causes of tropical lymphedema related morbidity in low resource settings [3]. Early-stage disease is characterized by mild symptoms, such as itching and reversible swelling of the lower leg [2, 4]. Later, acute dermato-lymphangio-adenitis (ADLA), fibrotic nodules and irreversible swelling arise [5]. Barefoot subsistence farmers and genetically susceptible individuals living at high altitudes are more likely to contract the disease [2]. Consistent footwear use and regular washing with soap and water are simple low-cost measures that protect against podoconiosis [6, 7].

Across Africa, 19 countries are considered endemic for podoconiosis [8, 9]. Only four countries have estimated national prevalence of the disease—Cameroon (0.5%), Ethiopia (7.45%), Rwanda (0.00068%) and Kenya (0.3%) [9–11]. For other endemic countries, prevalence estimates are based on small focal studies [9]. The East African region has the highest environmental probability for podoconiosis occurrence and is home to 81.7% of the 114.5 million people at risk of developing podoconiosis [12]. Geographical overlap between podoconiosis and lymphatic filariasis has also been highly predicted among East African countries [12]. People living with podoconiosis experience reduced quality of life as a result of restricted community participation, reduced productivity, discrimination, marginalization, low marriage prospects, psycho-social problems (depression, stress, and anxiety) and chronic poverty [13–15].

With early diagnosis and treatment, podoconiosis is reversible [6]; however, misperceptions regarding etiology and prevention exist and contribute to late or incorrect diagnoses and poor patient outcomes [16–18]. In Ethiopia, less than one-third of healthcare professionals reported adequate knowledge and skills to successfully treat podoconiosis patients [19]. Health care workers in Rwanda also exhibited low awareness of key diagnostic criteria and at-risk groups for podoconiosis [20]. Other studies in Ethiopia revealed that patients often discontinued treatment due to lack of adequate information on effectiveness or fear of being stigmatized by health workers [19, 21, 22]. An evaluation of podoconiosis education in medical schools found that only 27 out of 63 (42.9%) of medical programs in endemic East African countries included podoconiosis content in their curricula [23].

Nurses play a major role in early detection, patient education, and disease management of NTDs [24, 25]. The dearth of physicians in many African countries has led to task shifting, with nurses taking greater responsibility for disease diagnosis and management, especially in rural and remote areas [26–28]. Therefore, it is critical that nurses possess correct information and skills to rectify patient and community misconceptions and give advice on appropriate prevention and management measures [1, 29, 30]. At present, it is unclear whether nursing curricula at East African post-secondary institutions provide adequate training on podoconiosis detection and management. The objectives of this study were to evaluate the quality and quantity of podoconiosis instruction, and to identify barriers to inclusion of podoconiosis content in nursing curricula in Kenya, Rwanda, and Uganda.

## Methods

### Study design

A cross-sectional, quantitative assessment of post-secondary nursing institutions was conducted between July and September 2020. The study was conducted across three East African countries in which podoconiosis is endemic—Kenya, Rwanda, and Uganda.

**Fig. 1:**
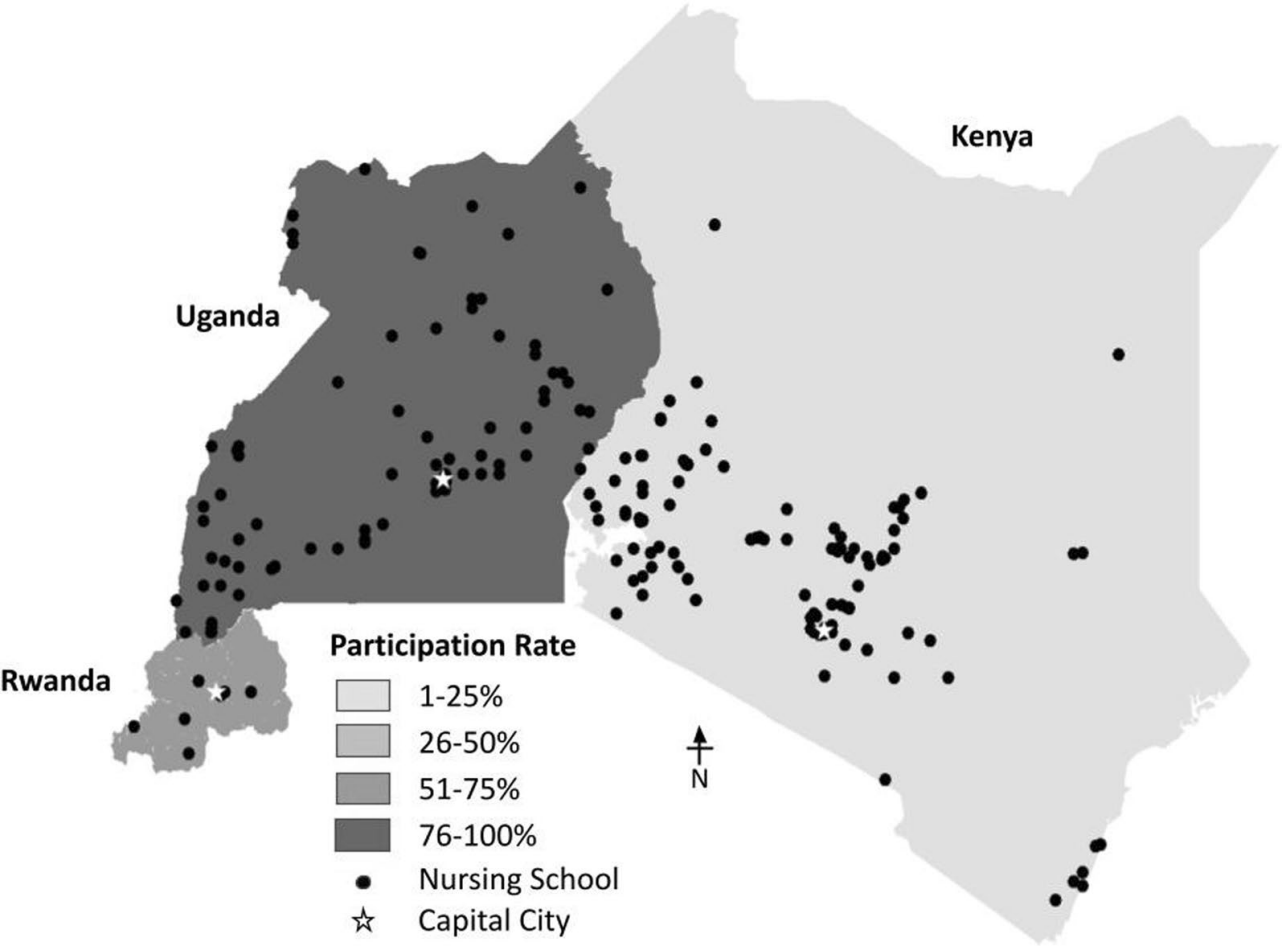
Geographic distribution and survey participation among accredited nursing institutions in Kenya, Rwanda, and Uganda (N = 149)

Through a rigorous online search strategy, we identified 86 accredited nursing certificate programs, 151 accredited nursing diploma programs and 57 accredited nursing degree programs across the three countries (Fig. 1). We examined country-level higher education documents, resources from international institutions, such as WHO and non-governmental higher education websites. We engaged nursing faculty to verify the list of eligible schools and ensure government accreditation.

### Data collection tool

Survey questions were initially developed by this team to assess podoconiosis instruction at African medical schools and were modified for nursing schools [23]. The survey tool was developed in English, pre-tested on 14 nursing tutors and lecturers in East Africa, and adapted as necessary. It was primarily comprised of multiple-choice questions but included several open-ended questions. Once ready, it was uploaded to an online collection platform (QualtricsXM) and made available in English. Completion of the survey took respondents 15–20 min.

Contact information for respondents was acquired by online searches of university websites, professional listings, LinkedIn and Research Gate; a thorough PubMed review of literature published by nursing schools to obtain author email addresses; exhaustive networking through the research team’s personal and professional academic and non-governmental organization contacts, and referrals by other respondents. Ultimately, we obtained contact information for 85 accredited nursing certificate programs, 148 accredited nursing diploma programs and 56 accredited nursing degree programs, and invited individuals from these programs to participate.

### Study population

Target respondents were principals/deans/department heads or other persons identified by the principal as knowledgeable about the institution’s nursing curriculum. For institutions that operated under umbrella organizations and using the same curricula, we approached a single curriculum expert from the administrative headquarters to provide representative information. The research team reached out to target participants through email, direct phone call, and/or messaging (if contact number was available). We supplemented the email invitations with formal invitation letters which provided the target participant with an overview of the research including contact numbers for the principal researchers in case of any questions. Data was collected in-person through telephone/Skype interviews or online through QualtricsXM. One member of the research team monitored the database for errors during the collection period.

### Statistical analysis

Study measures focused on the quantity of podoconiosis content, perceptions regarding the quality of podoconiosis education, and barriers preventing sufficient podoconiosis education. Knowledge of curricula was self-reported and perspectives were based on the respondents’ own perceptions of the Likert scale. Two researchers (LW, KF) independently carried out descriptive statistical analysis on all quantitative variables using SPSS (IBM, V.23) and R+, and compared results to ensure rigor of findings. Frequencies were used to summarize categorical variables, while median and range were used to describe the continuous variables. Open-ended questions were analyzed following the simple Hotjar analysis steps, wherein respondents’ opinions and experiences with podoconiosis were categorized into themes by LW and JMS [32].

## Results

Of the 228 faculty/staff invited to participate, 109 individuals gave information about 212 programs across 149 nursing institutions (response rate: 73.4%) across three countries. Most participants responded online (83.5%) versus by telephone (16.5%). Response rates were variable, ranging from 22.7% in Kenya to 71.4% in Rwanda and 96.6% in Uganda (Table 1). Reasons for non-participation included institutional protocols prohibiting participation, desire to be compensated, and lack of time.

**Table 1:** Accredited post-secondary programs in human nursing across Kenya, Rwanda and Uganda

Program	# Accredited nursing programs	# Invited	Participated n (%)
Kenya			
Certificate	9	9	9 (100)
Diploma	92	89	47 (52.8)
Degree	32	32	12 (37.5)
Rwanda			
Certificate	0	0	0 (0)
Diploma	3	3	2 (66.7)
Degree	4	4	3 (75.0)
Uganda			
Certificate	77	76	70 (92.1)
Diploma	56	56	50 (89.3)
Degree	21	20	19 (95.0)
Total	294	289	212 (73.4)

Study participants were predominantly female (60.6%), aged 40–49 years (35.8%), had earned a master’s degree (52.3%), and were principals/deans (57.8%) at their institution (Table 2). Many had heard about podoconiosis before (52.3%), knew that podoconiosis was caused by contact with soil (68.6%), and some associated the disease with poverty (5.88%). Nearly one in five answered incorrectly that podoconiosis was caused by mosquitoes (17.6%).

**Table 2:** Respondent demographics and podoconiosis knowledge (N = 109)

	Kenya (n = 20)	Rwanda (n = 3)	Uganda (n = 86)	Total (N = 109)
	Count (%)			
Gender				
Female	11 (55.0)	0 (0)	55 (64.0)	66 (60.6)
Male	9 (45.0)	3 (100)	31 (36.0)	43 (39.4)
Age (years)				
20–29	0 (0)	0 (0)	2 (2.33)	2 (1.83)
30–39	8 (40.0)	1 (33.3)	16 (18.6)	25 (22.9)
40–49	4 (20.0)	2 (66.7)	33 (38.4)	39 (35.8)
50–59	4 (20.0)	0 (0)	20 (23.3)	24 (22.0)
≥ 60	3 (15.0)	0 (0)	15 (17.4)	18 (16.5)
Prefer not to say	1 (5.0)	0 (0)	0 (0)	1 (0.92)
Highest qualification earned				
Advanced diploma	1 (5.0)	0 (0)	9 (10.5)	10 (9.2)
Undergraduate degree	2 (10.0)	0 (0)	32 (37.2)	34 (31.2)
Master’s degree	12 (60.0)	2 (66.7)	43 (50.0)	57 (52.3)
PhD degree	5 (25.0)	1 (33.3)	2 (2.30)	8 (7.30)
Academic title				
Lecturer	3 (15.0)	0 (0)	6 (7.0)	9 (8.30)
Professor	0 (0)	0 (0)	1 (1.16)	1 (0.90)
Administrator	0 (0)	1 (33.3)	10 (11.6)	11 (10.1)
Department Head	8 (40.0)	0 (0)	17 (8.10)	25 (22.9)
Dean	9 (45.0)	2(66.7)	52(60.5)	63(57.8)
Had ever heard of podoconiosis^a^	15 (75.0)	3 (100)	39 (45.3)	57 (52.3)
Aware that podoconiosis is endemic to their country				
Yes	2 (18.2)	1 (50.0)	16 (48.5)	19 (41.3)
No	9 (82.8)	1 (50.0)	17 (51.5)	27 (58.7)
Missing	4	1	6	11
Podoconiosis etiology				
Soil	10 (71.4)	2 (66.6)	23 (67.6)	35 (68.6)
Poverty	0 (0)	0 (0)	3 (8.82)	3 (5.88)
Hereditary	0 (0)	1 (33.3)	0 (0)	1 (1.96)
Mosquitoes	4 (28.6)	0 (0)	5 (14.7)	9 (17.6)
Randomly occurring	0 (0)	0 (0)	2 (5.88)	2 (3.92)
Direct contact	0 (0)	0 (0)	1 (2.94)	1 (1.96)
Population at high risk of developing podoconiosis				
Farmers	10 (100)	3 (100)	29 (93.5)	42 (95.5)
Children	0 (0)	0 (0)	2 (6.45)	2 (4.55)
Truck drivers	0(0)	0 (0)	0 (0)	0 (0)
Other	0 (0)	0 (0)	0 (0)	0 (0)
Effective preventive measures^b^				
Wearing shoes	9 (64.3)	3 (100)	20 (54.1)	32 (59.3)
Washing daily	9 (64.3)	2 (66.7)	19 (51.4)	30 (55.6)
Healthy diet	2 (14.3)	0	0	2 (3.70)
Mosquito control	4 (2.86)	0	3 (8.12)	7 (13.0)
Other^c^	0	0	10 (27.0)	10 (18.5)

^a^Respondents who had never heard of podoconiosis were not asked remaining knowledge questions

^b^Respondents could choose more than one option

^c^Other responses included covering holes in the home, avoiding rivers/lakes, treating people who live near rivers/lakes, health education, checking the soil for irritants, and staying in a clean environment

Most respondents (95.5%) correctly identified farmers as the highest risk group. Knowledge of shoe wearing (59.3%) and daily skin hygiene (55.6%) to prevent the disease were less well understood. Other preventative measures mentioned by respondents included maintaining a clean environment, health education, soil surveillance, reducing contact with red soil, and using floor coverings. Incorrect prevention strategies included mosquito control, ingesting cetirizine tablets and avoiding water bodies. Of the 52.3% who had heard about podoconiosis, 58.7% were not aware that it was endemic in their country. The average time required to earn nursing credentials varied by program and country (Table 3), ranging from 1.5 years for an extension diploma in Uganda to 5 years for a degree in Kenya.

**Table 3:** Program characteristics of government-accredited post-secondary institutions offering nursing programs in podoconiosis-endemic countries in East Africa (N = 149)

	Kenya (n = 60)	Rwanda (n = 3)	Uganda (n = 86)	Total (N = 149)
	Count (%)			
Program length in years, median (range)				
Certificate	2.5 (2.5–2.5)	n/a	2.5 (2.5–2.5)	2.5 (2.5–2.5)
Diploma	3.5 (3.0–3.5)	3 (3–3)	3 (2–3)	3.5(2–3.5)
Degree	4 (4–5)	4 (4–4)	4 (2.5–5)	4 (2.5–5)
# graduates/year, median (range)				
Certificate	800 (800–800)	n/a	85 (12–600)	87.5 (5–800)
Diploma	50 (20–4500)	55 (50–60)	20 (5–175)	30 (5–4500)
Degree	50 (25–150)	70 (70–70)	32.5 (10–200)	45 (10–200)
Includes courses specific to NTDs^a^, n (%)				
Certificate	9 (100)	n/a	48 (68.6)	57 (72.2)
Diploma	45 (95.7)	2 (100)	32 (64.0)	79 (79.8)
Degree	11 (91.7)	2 (66.6)	10 (20.8)	23 (69.7)
Offers podoconiosis training, n (%)				
Certificate	8 (88.9)	n/a	11 (15.7)	19 (24.1)
Diploma	41 (87.2)	1 (50.0)	13 (26.0)	55 (55.6)
Degree	2 (16.7)	2(66.7)	6 (33.3)	10 (30.3)
Offers podoconiosis training to CHWs^b^, n (%)	1 (5.56)	0 (0)	3 (3.75)	4 (3.90)
Location				
Urban	8 (40.0)	0 (0)	47 (54.7)	55 (50.5)
Rural	11 (55.0)	3 (100)	39 (45.3)	53 (48.6)
I don’t know	1 (5.0)	0 (0)	0 (0)	1 (0.90)

*n/a* not applicable in Rwanda as it does not offer human nursing programs at certificate level

^a^Neglected Tropical Diseases

^b^Community Health Workers are individuals with no formal medical education who are elected to support basic health services in their communities

The annual number of nursing graduates ranged from 12 for nursing certificates in Uganda to 4500 for a nursing diploma in Kenya. More than three quarters (81.7%) of the nursing schools also offered programs in midwifery; some offered medicine and public health (15.6%). NTD-specific courses were offered in most of the programs (minimum—69.7%, maximum—79.8%), but provision of podoconiosis training was much lower (minimum—24.1%, maximum—55.6%). Overall, 48.6% of the nursing programs included in the study were rural; only 3.9% taught Community Health Workers (CHWs). Podoconiosis training varied considerably by program and by country (Table 4). In Kenya, NTD courses were largely integrated in curricula (> 90.0%), but podoconiosis-specific instruction was only common in certificate (88.9%) and diploma (87.2%) programs, occurring mostly during clinical rotations. The median time reported for preclinical podoconiosis instruction was 4 h; attendance was mandatory. Most respondents reported that their students were likely to interact with podoconiosis patients during clinical training (ranging from 66.9% to 100%). Approximately half (55.0%) of nursing programs were based in rural areas and 5.6% offered podoconiosis training to CHW’s. Rwanda did not offer nursing certificates. Two-thirds of degree and all diploma programs offered courses specific to NTDs; however, podoconiosis-specific training was more often reported in degree programs (66.6%). No specific amount of time was dedicated to teaching podoconiosis, but podoconiosis-specific instruction was offered both during pre-clinical coursework and/or during clinical rotations; attendance was mandatory. All respondents reported that their students were likely to interact with podoconiosis patients during their clinical attachments. All programs were rural and none offered podoconiosis training to CHWs. In Uganda, courses specific to NTDs were commonly offered for certificate (68.6%) and diploma (64.0%) programs, less in degree programs (20.8%). Podoconiosis training was reported in up to one-third of programs (certificate—15.7%, diploma—26.0%, degree—33.3%). Of the schools that offered podoconiosis-specific courses, instruction was often incorporated in pre-clinical and clinical training; and attendance for all classes was mandatory. Irrespective of program, most respondents reported that students were likely to interact with podoconiosis patients during their clinical attachments. Overall, 45.3% of programs were rural; only 3.8% offered training to CHWs.

**Table 4:** Inclusion of podoconiosis content in nursing program curricula and perceived importance and sufficiency of podoconiosis training offered across nursing programs (N = 212)

	Kenya (n = 68)				Rwanda (n = 5)			Uganda (n = 139)			
	Certificate	Diploma	Degree	Total	Diploma	Degree	Total	Certificate	Diploma	Degree	Total
Any podoconiosis training, n (%)	8 (88.9)	41 (87.2)	2 (16.7)	51 (75.0)	1 (50)	2 (66.7)	3 (60.0)	11 (15.7)	13 (26)	6 (33.3)	30 (21.6)
Pre-clinical podoconiosis training^b^, n (%)	0 (0)	0 (0)	1 (50.0)	1 (1.96)	0 (0)	0 (0)	0 (0)	9 (81.8)	11 (84.6)	4 (66.7)	24 (80.0)
Pre-clinical lecture hours, median (range)	0 (0)	0 (0)	4 (4–4)	4 (4)	0 (0)	0 (0)	0 (0)	1.75 (0.9–10)	2.0 (0.9–48)	8.0 (2–8)	2 (0.9–48.0)
Clinical podoconiosis training^c^, n (%)	8 (100)	39 (95.1)	2 (100)	49 (96.1)	0 (0)	1 (50.0)	1 (33.3)	6 (66.7)	9 (69.2)	4 (66.7)	19 (63.3)
Interact with podoconiosis patients, n (%)	8 (88.9)	43 (93.5)	7 (70.0)	58 (89.2)	2 (100)	3 (100)	5 (100)	44 (65.7)	35 (71.4)	10 (58.8)	89 (66.9)
Training quality											
Very sufficient	1 (50.0)	2 (22.2)	2 (18.2)	5 (22.7)	0 (0)	0 (0)	0 (0)	3 (4.3)	2 (4.0)	1 (5.3)	6 (4.3)
Somewhat sufficient	0 (0)	1 (11.1)	1 (9.09)	2 (9.09)	0 (0)	1 (33.3)	1 (20)	3 (4.3)	5 (10.0)	1 (5.3)	9 (6.47)
Undecided	0 (0)	1 (11.1)	3 (27.3)	4 (18.2)	1 (50)	1 (33.3)	2 (40)	9 (12.9)	10 (20)	6 (31.6)	25 (18)
Somewhat insufficient	0 (0)	0 (0)	3 (27.3)	3 (13.6)	0 (0)	0 (0)	0 (0)	12 (17.1)	11 (22)	5 (26.3)	28 (20.1)
Very insufficient	1 (50.0)	5 (55.6)	2 (18.2)	8 (36.4)	1 (50)	1 (33.3)	2 (40)	43 (61.4)	22 (44)	6 (31.6)	71 (51.1)
Training quantity											
Very sufficient	1 (50)	2 (22.2)	1 (7.7)	4 (18.2)	0	0	0	2 (2.86)	3 (6)	2 (10.5)	7 (5.0)
Somewhat sufficient	0	0	1 (7.7)	1 (4.65)	0	1 (33.3)	1 (20.0)	5 (7.14)	3 (6.0)	2 (10.5)	10 (7.2)
Undecided	0	1 (11.1)	6 (46.2)	7 (31.8)	1 (50)	1 (33.3)	2 (40)	7 (10.0)	11 (22)	5 (26.3)	23 (16.5)
Somewhat insufficient	0	1 (11.1)	1 (7.7)	2 (9.1)	0	0	0	16 (22.9)	11 (22)	2 (10.5)	29 (20.9)
Very insufficient	1 (50)	5 (55.6)	4 (30.8)	9 (45.5)	1 (50)	1 (33.3)	2 (40)	43 (61.4)	22 (44)	8 (42.1)	70 (50.4)

Across the three countries, participants between (36.4–51.1%) and (45.5–50.4%) felt that the quality and quantity, respectively, was very insufficient. Furthermore, (13.6–20.1%) and (9.1–20.9%) felt that the quality and quantity, respectively, was somewhat insufficient. Programs that offered podoconiosis training during pre-clinical training often integrated it in courses, such as tropical medicine (*n* = 11), community health (*n* = 8), microbiology (*n* = 7), epidemiology (*n* = 2), communicable diseases (*n* = 2) and/or parasitology (*n* = 2). Those offering it during clinical training included it during medical surgical nursing (*n* = 4) and pathophysiology (*n* = 1) rotations. The most cited barriers to providing podoconiosis training across all programs in the three countries were: not being part of the government curriculum (53.3%), the disease being of low priority (38.3%), and low faculty knowledge (24.3%; Table 5).

**Table 5:** Perceived barriers to inclusion of podoconiosis content in nursing curriculum and ranked importance of podoconiosis training (N = 108)

	Kenya (n = 19)	Rwanda (n = 3)	Uganda (n = 86)	Total (N = 108)
	Count (%)			
Podoconiosis training barriers				
Lack time	3 (15.8)	0 (0)	8 (9.4)	11 (10.3)
Insufficient equipment	1 (5.3)	0 (0)	3 (3.50)	4 (3.8)
Lack funding	1 (5.3)	0 (0)	8 (9.40)	9 (8.4)
Not in government curriculum	6 (31.6)	1 (33.3)	50 (58.8)	57 (53.3)
Low faculty knowledge	3 (15.8)	0 (0)	23 (27.1)	26 (24.3)
Low priority	10 (52.6)	2 (66.6)	29 (34.1)	41 (38.3)
Other^a^	0 (0)	0 (0)	6 (7.1)	6 (5.6)
None	2 (10.5)	0 (0)	0 (0)	2 (1.9)
Ranked importance of podoconiosis training				
High	4 (21.1)	2 (66.6)	39 (45.3)	45 (41.7)
Moderate	10 (52.6)	1 (33.3)	20 (23.3)	31 (28.7)
Low	5 (26.3)	0 (0)	27 (31.4)	32 (29.6)

^a^Other responses included lack of adequate research on podoconiosis

Other barriers included lack of research on podoconiosis, rarity of patients and lack of resources (equipment, time, and funding). Respondents mentioned that content delivered on various nursing programs was pre-determined in the approved government curricula. Government curricula related to NTDs focused on more common ailments, such as lymphatic filariasis, trypanosomiasis, and schistosomiasis.

“It's not part of the nursing curriculum and students do not waste time on matters that will not be examined” (ID_31).

“Neglected diseases are all neglected while teaching. We tend to stick to common diseases specified in the curriculum. Since the curriculum is designed by the government any lecturer with the knowledge may bring it to learners as merely a nice to know” (ID_25).

Among the few schools that addressed podoconiosis, respondents mentioned that their teaching was not well grounded in context. Some respondents expressed difficulties in teaching a topic that they themselves were not taught. Moreover, absence of adequate research on podoconiosis, coupled with overwhelming workload, further complicated any effort to introduce podoconiosis content.

“It’s sometimes hard to teach as myself I have never seen one. You cannot teach with mere imaginations, and you don’t have the confidence to talk about it.” (ID_60).

Overall, respondents who ranked the importance of podoconiosis training as low or moderate were almost even (29.6% v 28.7%). Common reasons given for low/moderate priority were (1) podoconiosis was rare (2) disease is not fatal and (3) patients do not seek healthcare services. Respondents explained that podoconiosis patients rarely seek medical attention, and therefore, students are less likely to encounter them during clinical rotations Nonetheless, some still felt that it was very easy to integrate podoconiosis into the curriculum for tropical diseases which many nursing programs already offered as a course.

“The condition is not common and the few clients with the condition never seek medical care (ID_103).

“I would think it's not common in our country and I believe the country has not taken keen interest in this condition.” (ID_46)

The remaining 41.7% of nursing faculty across all three countries felt that providing podoconiosis training for nurses was a high priority. Common reasons for this ranking included (1) the need for differential diagnosis between lymphatic filariasis and podoconiosis, (2) the importance of delivering equitable health care, (3) the low cost of podoconiosis prevention relative to treatment, (4) the need for nurses to work in rural communities, where the disease is more prevalent, and (5) the devastating consequence of podoconiosis at late stages.

“Podoconiosis is a rare condition but should be integrated in nursing cadres that are likely to interact closely with communities, such as certificate nurses” (ID_88).

Rural populations, particularly farmers and those living in extreme poverty, were highlighted as most-at-risk for developing podoconiosis. Respondents also agreed with the benefit of having a comprehensive curriculum but noted that there are very educational activities that serve to increase podoconiosis specific knowledge and skills among nursing tutors.

“We have a challenge that we have limited CME’s [*Continuous Medical Education*] so there is a gap. Hospitals need to fill this gap” (ID_45).

## Discussion

The latest WHO NTD Roadmap calls for a 75% reduction in disability adjusted life-years associated with NTDs, including podoconiosis, by 2030 [31]. This study of nursing curricula in Kenya, Rwanda, and Uganda, aligns with a previous evaluation of medical curricula across 19 endemic countries, demonstrating widespread training gaps, with limited coverage for podoconiosis content across programs. It helps to explain the current challenges, including patient stigma and inappropriate treatments, in managing podoconiosis patients in East Africa [6, 19, 20]. With many vulnerable communities still experiencing shortages in human resources for health and unable to routinely diagnose and report podoconiosis [8], there is an urgent need to evaluate and improve resources available for training nurses and physicians working in endemic areas. Altogether, this study highlights the need for podoconiosis training materials that are freely available, contextually appropriate, and accessible to both clinical instructors and those already in practice.

We identified knowledge gaps in podoconiosis etiology and prevention among nursing faculty, even though many respondents had heard of the disease and seen affected patients in clinics. This is likely because most respondents had not received formal training on podoconiosis when completing their own nursing education. Elsewhere, there is scarcity of reliable and comprehensive information on podoconiosis [9, 10], including research on interventions [33]. As a result, important information on disease trends, risk factors, disease burden and patterns of care remains largely uncollated and poorly known. Our findings align with previous studies from Rwanda and Ethiopia demonstrating that health workers lacked the specific knowledge to effectively treat and care for podoconiosis patients [19–21, 34]. Similar misperceptions on disease cause and management were documented among medical faculty in endemic Sub-Saharan countries [23]. Altogether, this highlights a shared need for podoconiosis education for nurses and physicians to accelerate early detection, reduce patient stigma, improve prevention, and optimize patient outcomes.

Nursing faculty felt that the quality and quantity of podoconiosis training across nursing programs was generally insufficient. This aligned with the medical school results, where most respondents felt that the quality (60.4%) and quantity (61.5%) of instruction was insufficient [23]. There is no substantive evidence to define how much podoconiosis content is sufficient in medical/nursing education; however, class-based learning could be improved through integrated teaching approaches [31]. For instance, podoconiosis could be incorporated in courses such as parasitology, tropical medicine, and community health as well as in pre-existing NTD courses particularly topics on lymphoedema or skin-NTDs. On the other hand, clinical work-placements could be prioritized over classroom study to enhance hands-on clinical skills [34, 35]. Hands-on programs could include supervised internships, practicums, and community outreaches. Clinical training improves understanding of processes and systems of healthcare delivery and shapes the correct attitudes among health professionals [36, 37]. Moreover, clinical experience was found to have a positive correlation with podoconiosis management and disability prevention [34]. The WHO also recommended inter-professional training as one way to build knowledge while gaining multi-disciplinary skills and maximizing resources [38]. Nurses could, therefore, be trained alongside students from other backgrounds, such as veterinary medicine, social sciences and environmental health for more comprehensive knowledge. This training could extend to community health aides, such as CHWs, community volunteers and community health educators who are already active and more likely to support the nurses’ work in the local community [25, 39–41].

Nurses are key in coordinating and delivering NTD care [24, 25], and they play a major role in directing health education, promotion, and disease control activities [25]. Even though podoconiosis is often not a priority for governments in endemic settings [2], 41.7% of the faculty in our study ranked the importance of podoconiosis training for nurses as high. As per [42], our respondents suggested that nurses were more likely to work in rural areas than physicians and should be prioritized for podoconiosis training. Curriculum reforms to include rare illnesses have long been recommended in Africa [43]; however, several challenges undermine inclusion for podoconiosis. These obstacles include lack of time, limited training space, inadequate faculty capacity and government policy. Learning from Ethiopia, it is critical to generate evidence about the distribution, burden and relative simplicity of preventive interventions, to increase visibility and prioritization of podoconiosis among policy makers and ultimately for curriculum inclusion [33, 44, 45]. Government support and commitment is essential to increase resources and infrastructure for podoconiosis education.

Despite the East African region being home to numerous podoconiosis endemic countries, our study was limited to three, and excluded highly endemic countries, such as Ethiopia. Even with an acceptable overall response rate (73.4%), the country-level rate ranged from 22.7 to 71.4%, due in part to school closures in response to the COVID-19 pandemic. These results should not be considered representative for the whole East African region. Second, our data relied on self-reported knowledge about institutional curriculum. We minimized the likelihood of inaccurate responses by sharing study objectives during recruitment and allowing participants to refer to the official curricula documents of their institution. Finally, our study only assessed podoconiosis education related to nursing students, but we acknowledge that there are healthcare cadres such as health officers and community health workers who play important roles in disease detection and health education. Despite these limitations, this study is the first to characterize podoconiosis education among nursing institutions in endemic East Africa.

## Conclusions

Nurses play a key role in early detection, case management, and community sensitization of podoconiosis. Addressing knowledge gaps among this cadre is an important step to achieving local, regional, and global goals for podoconiosis prevention and management. Developing and sharing regionally appropriate learning materials for healthcare professionals and health educators could substantially elevate the care received by vulnerable populations, especially in rural areas, and could contribute to burden reduction.

## Data Availability

The data sets used and/or analyzed during the current study are available from the corresponding author on reasonable request.

## Abbreviations

NTDs: Neglected Tropical Diseases
WHO: World Health Organization
ADLA: Acute dermato-lymphangio-adenitis
CHWs: Community Health Workers
